# Histone acetyltransferase GCN5 orchestrates flower development and is required for proper regulation of multiple key meristem and organ identity genes

**DOI:** 10.1093/plcell/koaf153

**Published:** 2025-06-12

**Authors:** Jiajun Wang

**Affiliations:** Assistant Features Editor, The Plant Cell, American Society of Plant Biologists; School of Life Sciences, Xiamen Key Laboratory of Plant Genetics, Xiamen University, Xiamen 361102, China

The development of floral organs, tightly regulated by precise spatiotemporal gene expression, enables angiosperms to reproduce through the formation of fruits and seeds, thereby ensuring species survival. Floral organogenesis begins when stem cells in the floral meristem (FM) undergo controlled differentiation to give rise to the characteristic concentric whorls of floral organs. The maintenance and termination of stem cell activity in the FM are crucial for proper floral development. The WUSCHEL-CLAVATA3 (WUS-CLV3) feedback loop maintains stem cell homeostasis during early floral stages (Lopes et al. 2021). As development progresses, AGAMOUS (AG) directly or indirectly represses *WUS* expression through KNUCKLES (KNU), a C2H2 zinc-finger protein, and terminates stem cell activity at the appropriate developmental stage, ensuring the proper formation of carpels and completion of floral development (Liu et al. 2011; Shang et al. 2021).

General Control Non-Depressible 5 (GCN5) encodes a conserved histone acetyltransferase that is required for a wide range of developmental processes in plants, including plant height and the proper development of flowers and leaves (Bertrand et al. 2003). GCN5 forms the SAGA (Spt-Ada-Gcn5-Acetyltransferase) complex together with the associated coactivator ALTERATION/DEFICIENCY IN ACTIVATION2 (ADA2). This complex is capable of acetylating multiple histone residues, thereby modulating chromatin structure and promoting gene transcription (Vlachonasios et al. 2021). The molecular mechanism by which GCN5 is needed for floral development is not well studied. In new work, **Amangul Hawar, Wei Chen, Tao Zhu, and collaborators (Hawar et al. 2025)** uncovered how GCN5 precisely orchestrates floral development in Arabidopsis (*A. thaliana*) by acting at multiple regulatory levels, including FM maintenance, FM determinacy, and the control of ABCE-class floral homeotic genes.

Through analysis of the *gcn5-7* mutant, the authors observed pronounced floral defects, including reduced carpel formation and abnormal stamen numbers. Among 150 flowers examined, 39 showed defects such as a single carpel, a replum-like cylindrical structure without carpels, or a complete absence of gynoecia. Stamen development was also disrupted, with 24 flowers producing more than 6 stamens and 29 flowers developing fewer than 6. To uncover the underlying mechanisms, the authors conducted transcriptome profiling and genome-wide H3K9Ac ChIP-seq on floral buds from both wild type and *gcn5-7*, along with Cleavage Under Targets & Tagmentation assays using *gcn5-7 pGCN5:GCN5-eGFP* floral buds to map the global binding sites of GCN5. These experiments revealed that GCN5 binds to the promoters of *WUS* and *CLV3* and promotes H3K9Ac enrichment across their gene bodies, enhancing their transcription. Notably, GCN5 also attenuates the ectopic expression of *WUS* in *clv3-2* and *knu-2* mutants. Compared to *clv3-2*, *gcn5-7 clv3-2* mutants showed a reduced number of carpels, while *gcn5-7 knu-2* mutants significantly suppressed the reiterative ectopic carpel phenotype characteristic of *knu-2* mutants.

To further explore how GCN5 is recruited to the *WUS* promoter and activates its transcription, the authors tested or screened for novel proteins that interact with GCN5 or ADA2b, a known companion protein of GCN5. Using yeast 2-hybrid assays, bimolecular fluorescence complementation, and pull-down assays, they identified interactions between ADA2b and the SWI/SNF chromatin remodeler SPLAYED (SYD), which modulates H3K27me3 levels through antagonistic or synergistic relationships with Polycomb group (PcG) proteins (Shu et al. 2021). Additionally, ADA2b interacted with the cytokinin-responsive B-type transcription factors ARR1 and ARR10. Subsequent yeast 3-hybrid assays, bimolecular luciferase complementation, and co-immunoprecipitation assays further demonstrated that ADA2b facilitates the formation of a SYD–ADA2b–ARR complex. More importantly, chromatin immunoprecipitation (ChIP)-quantitative PCR assays revealed that the binding of GCN5-eGFP to the *WUS* promoter was markedly reduced in both *syd-5* and *arr1 arr10* mutants. Consistently, *WUS* transcript levels were significantly decreased in both *syd-5* and *arr1 arr10 arr12* triple mutants. The dual-luciferase reporter assays further demonstrated that while GCN5 alone lacks *WUS* promoter activation capability, it potentiates ARR12-mediated transcriptional activation. Together, these findings suggest that ARRs recruit the GCN5–ADA2b–SYD ternary complex to the *WUS* promoter, with both GCN5 and SYD being essential for proper *WUS* expression.

Beyond its role in regulating FM activity through *WUS* and *CLV3*, GCN5 also controls FM determinacy by activating the expression of key regulators. Similar to its action at the *WUS* promoter, GCN5 binds to the promoters of *AG*, *KNU*, *CRC*, and *SUP*, with H3K9Ac levels at these loci significantly reduced in the *gcn5-7* mutant. Mechanistically, the authors identified an interaction between ADA2b and the transcription factor PERIANTHIA (PAN), suggesting that PAN recruits GCN5 to the *AG* promoter, as evidenced by markedly reduced GCN5 binding in *pan* mutants. Moreover, DEX-induced expression of *35S:AG-GR* in the *gcn5-7* background substantially rescued the reduced carpel phenotype, underscoring the functional importance of GCN5-mediated *AG* activation.

Notably, GCN5's regulatory scope extends across multiple floral homeotic gene classes. In addition to modulating the C-class gene *AG*, GCN5 also regulates A-class (*AP2*), B-class (*AP3*), and E-class (*SEP4*) gene expression. In *gcn5-7* mutants, *AP3* expression was significantly upregulated, and RNAi-mediated suppression of *AP3* in the *gcn5-7* background led to a marked reduction in stamen number, demonstrating GCN5's role in maintaining proper floral organ specification.

This work reveals that GCN5 orchestrates floral development through 2 functionally distinct complexes: the GCN5-ADA2b-SYD-ARRs module maintains FM activity by activating *WUS* expression, while the GCN5-ADA2b-PAN complex ensures FM determinacy via *AG* regulation. Additionally, GCN5 contributes to the proper regulation of floral development by modulating the expression of multiple genes such as *KNU*, *CRC*, *SUP*, and *AP3* (Figure).

**Figure.**
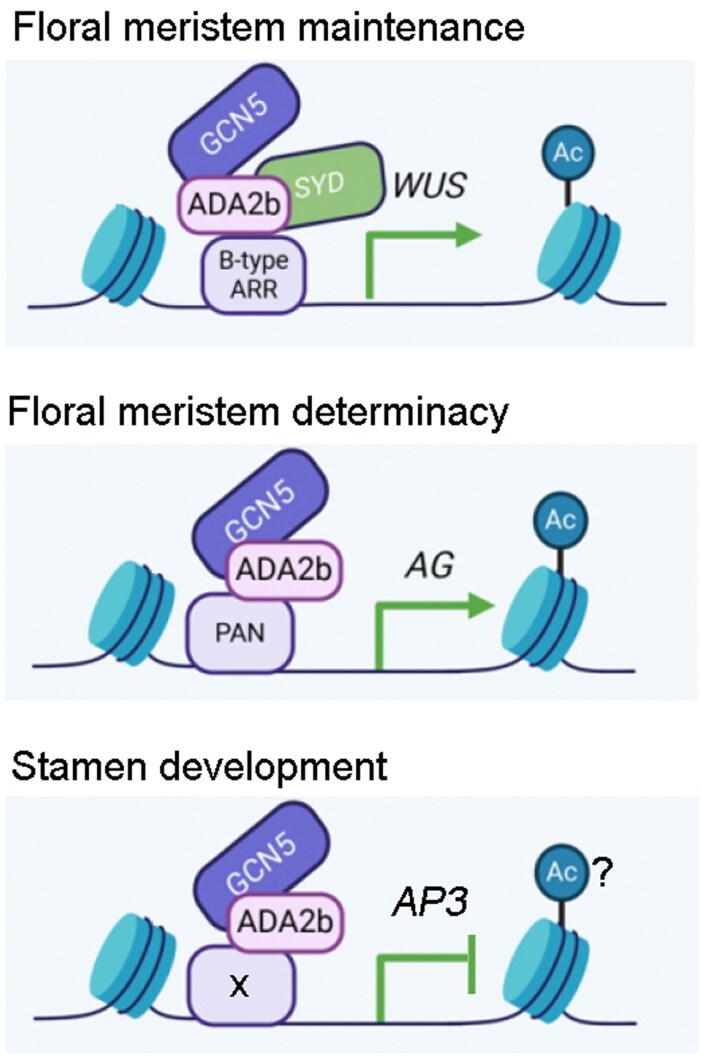
Model of GCN5-mediated regulation of floral development. During early floral development, the GCN5–ADA2b–SYD–ARRs complex is recruited to the *WUS* promoter to promote *WUS* expression, thereby maintaining FM activity. During floral organogenesis, the GCN5–ADA2b–PAN complex binds to the *AG* promoter to activate *AG* expression, which terminates FM activity. In addition, GCN5 suppresses the expression of *AP3* to regulate stamen development. Modified from Hawar et al. (2025), Supplementary Fig. S13.

## Recent related articles in *The Plant Cell*

- Pelayo et al. (2023) reported that AGAMOUS (AG) orchestrates FM termination and stamen development through a cell cycle-coupled H3K27me3 dilution timer, which temporally regulates downstream targets (including KNU, AHL18, and PLATZ10) by erasing repressive marks at different rates proportional to their H3K27me3-marked region lengths.
- Wang et al. (2024) demonstrated that stage-specific FM termination requires KNU-mediated H3K27me3 deposition at PIN1 and IPT7 loci, linking transcriptional repression of auxin/cytokinin pathways to stem cell niche inactivation.
- Hugouvieux et al. (2024) found that SEPALLATA3 tetramerization is crucial for both FM determinacy and organ identity specification, underscoring the importance of MADS complex formation in flower development.
- Bowman and Moyroud. (2024) reviewed the evolution and impact of the ABC model, summarizing key discoveries and unresolved questions in floral organ development.

## Dive Curated Terms

The following phenotypic, genotypic, and functional terms are of significance to the work described in this paper:

ARR1 Gramene: AT3G16857
ARR1 Araport: AT3G16857

PIN1 Gramene: AT1G73590
PIN1 Araport: AT1G73590

WUS Gramene: AT2G17950
WUS Araport: AT2G17950

CLV3 Gramene: AT2G27250
CLV3 Araport: AT2G27250

SUP Gramene: AT3G23130
SUP Araport: AT3G23130

SEP4 Gramene: AT2G03710
SEP4 Araport: AT2G03710

pan Gramene: AT1G68640
pan Araport: AT1G68640

ARR10 Gramene: AT4G31920
ARR10 Araport: AT4G31920

AP3 Gramene: AT3G54340
AP3 Araport: AT3G54340

crc Gramene: AT1G69180
crc Araport: AT1G69180

AP2 Gramene: AT4G36920
AP2 Araport: AT4G36920
